# Phantom Study Investigating the Accuracy of Manual and Automatic Image Fusion with the GE Logiq E9: Implications for use in Percutaneous Liver Interventions

**DOI:** 10.1007/s00270-017-1607-3

**Published:** 2017-02-15

**Authors:** Mark Christiaan Burgmans, J. Michiel den Harder, Philippa Meershoek, Nynke S. van den Berg, Shaun Xavier Ju Min Chan, Fijs W. B. van Leeuwen, Arian R. van Erkel

**Affiliations:** 1 Department of Radiology, Leiden University Medical Centre, Albinusdreef 2, 2300 RC Leiden, The Netherlands; 2 Interventional and Molecular Imaging Laboratory, Department of Radiology, Leiden University Medical Center, Leiden, The Netherlands; 3 Department of Interventional Radiology, Singapore General Hospital, Outram Road, Singapore, 169608 Singapore

**Keywords:** Phantom study, Liver interventions, Image fusion, Volume navigation, Co-registration methods

## Abstract

**Purpose:**

To determine the accuracy of automatic and manual co-registration methods for image fusion of three-dimensional computed tomography (CT) with real-time ultrasonography (US) for image-guided liver interventions.

**Materials and Methods:**

CT images of a skills phantom with liver lesions were acquired and co-registered to US using GE Logiq E9 navigation software. Manual co-registration was compared to automatic and semiautomatic co-registration using an active tracker. Also, manual point registration was compared to plane registration with and without an additional translation point. Finally, comparison was made between manual and automatic selection of reference points. In each experiment, accuracy of the co-registration method was determined by measurement of the residual displacement in phantom lesions by two independent observers.

**Results:**

Mean displacements for a superficial and deep liver lesion were comparable after manual and semiautomatic co-registration: 2.4 and 2.0 mm versus 2.0 and 2.5 mm, respectively. Both methods were significantly better than automatic co-registration: 5.9 and 5.2 mm residual displacement (*p* < 0.001; *p* < 0.01). The accuracy of manual point registration was higher than that of plane registration, the latter being heavily dependent on accurate matching of axial CT and US images by the operator. Automatic reference point selection resulted in significantly lower registration accuracy compared to manual point selection despite lower root-mean-square deviation (RMSD) values.

**Conclusion:**

The accuracy of manual and semiautomatic co-registration is better than that of automatic co-registration. For manual co-registration using a plane, choosing the correct plane orientation is an essential first step in the registration process. Automatic reference point selection based on RMSD values is error-prone.

## Introduction

Image guidance using ultrasonography (US) offers important advantages over computed tomography (CT) guidance for targeting of liver lesions during minimally invasive procedures such as biopsies and percutaneous ablations [1]. US allows real-time imaging, is not associated with radiation and offers the interventional radiologist a free choice of plane for needle placement. However, up to one-fifth of liver lesions are inconspicuous on US [2].

US systems with fusion imaging are commercially available from different vendors [3–6]. Three-dimensional (3D) computed tomography (CT) or magnetic resonance (MR) image data can be acquired before the intervention and uploaded onto these US systems for image fusion with real-time US images, using an electromagnetic transmitter and electromagnetic sensors attached to the transducer [7, 8]. To the interventional radiologist, the fusion imaging technology may be of great value as it allows targeting of lesions that are inconspicuous on US with reduced radiation exposure. Several clinical studies have demonstrated the usefulness of US-CT/MRI image fusion in targeting liver tumors that are inconspicuous on US [1–6].

For safe and accurate use of these navigation systems, accurate matching (co-registration) of the 3D image datasets with the real-time US images is essential. Inaccuracies in co-registration may lead to technical failure or inadvertent ablation of healthy liver tissue. Co-registration can be performed either manually or automatically. Manual co-registration requires indication of reference points or planes by the operator in the real-time US data and their corresponding positions or planes in the 3D dataset [9, 10]. It can be challenging, requires experience and does not compensate for patient movement. A variable learning curve is experienced for obtaining consistent and accurate manual co-registration. Automatic co-registration by the ultrasound system on the other hand makes use either of automatic image recognition or of a frame with fiducial markers, attached to the patient’s body [11, 12]. Automatic co-registration saves time, can compensate for patient movement and is feasible even if ultrasonographic visualization of the liver is compromised, due to, e.g., obesity, overlying air, steatosis or cirrhosis. Though automatic co-registration offers an easier to use and learn platform than manual co-registration, the accuracy of automatic registration has not been determined.

In this study, we compared the accuracy of manual and automatic co-registration for liver lesions in a phantom. Additional experiments demonstrate the benefits and caveats of different manual co-registration methods. Based on experiments, we aim to provide recommendations for efficient, reliable and accurate co-registration.

## Materials and Methods

### Equipment

**Fig. 1:**
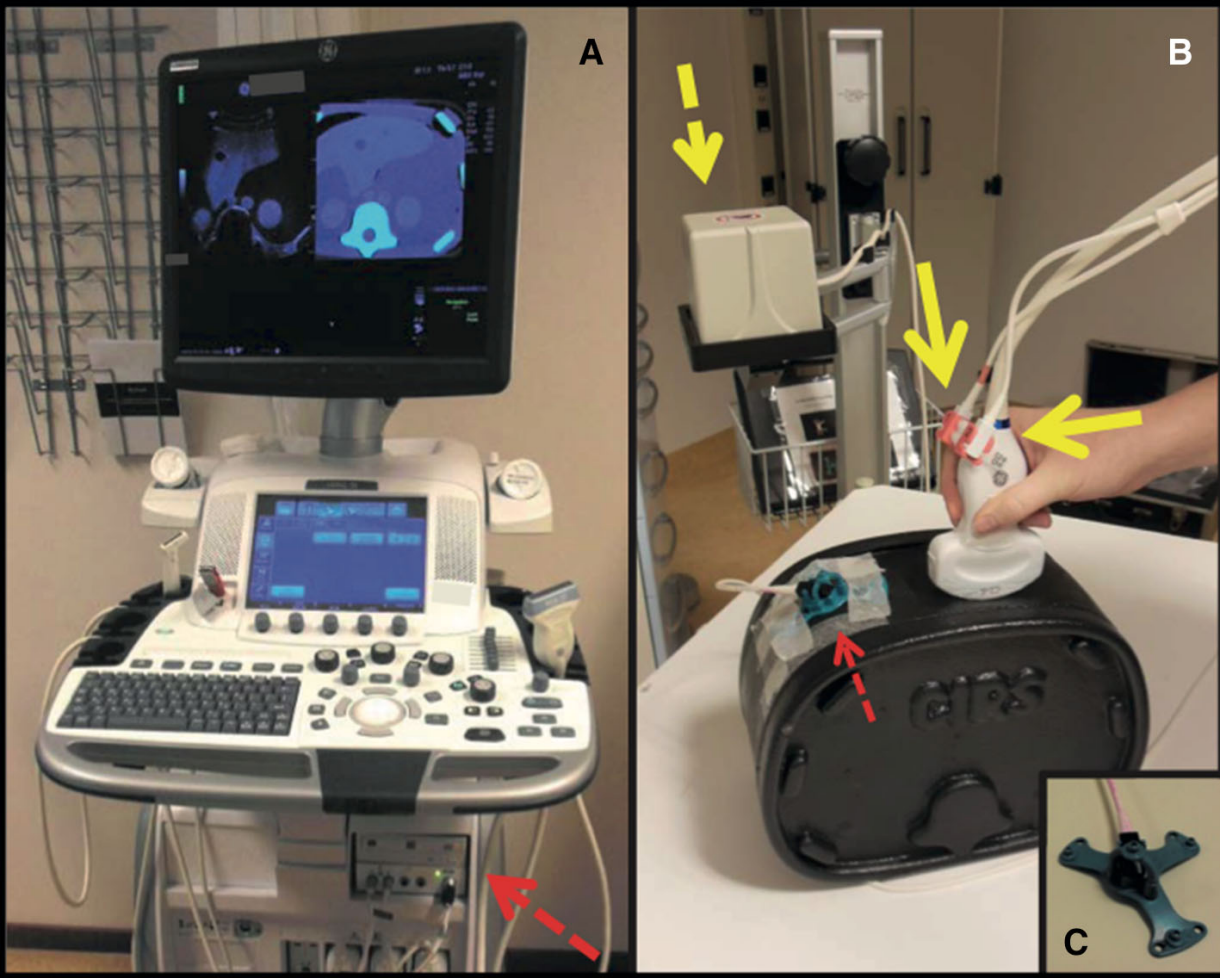
Volume navigation system and phantom setup: **A** GE Logiq E9 US system with volume navigation module (*dashed arrow*). **B** C1-6-D convex transducer equipped with two electromagnetic sensors (*solid yellow arrows*). The electromagnetic transmitter is positioned next to the phantom (*yellow dashed arrow*) and the OmniTRAX™ Active Patient Tracker attached to the phantom (*red dashed arrow*). **C** OmniTRAX™ Active Patient Tracker with four radio-opaque fiducial markers and an additional electromagnetic sensor

**Fig. 2:**
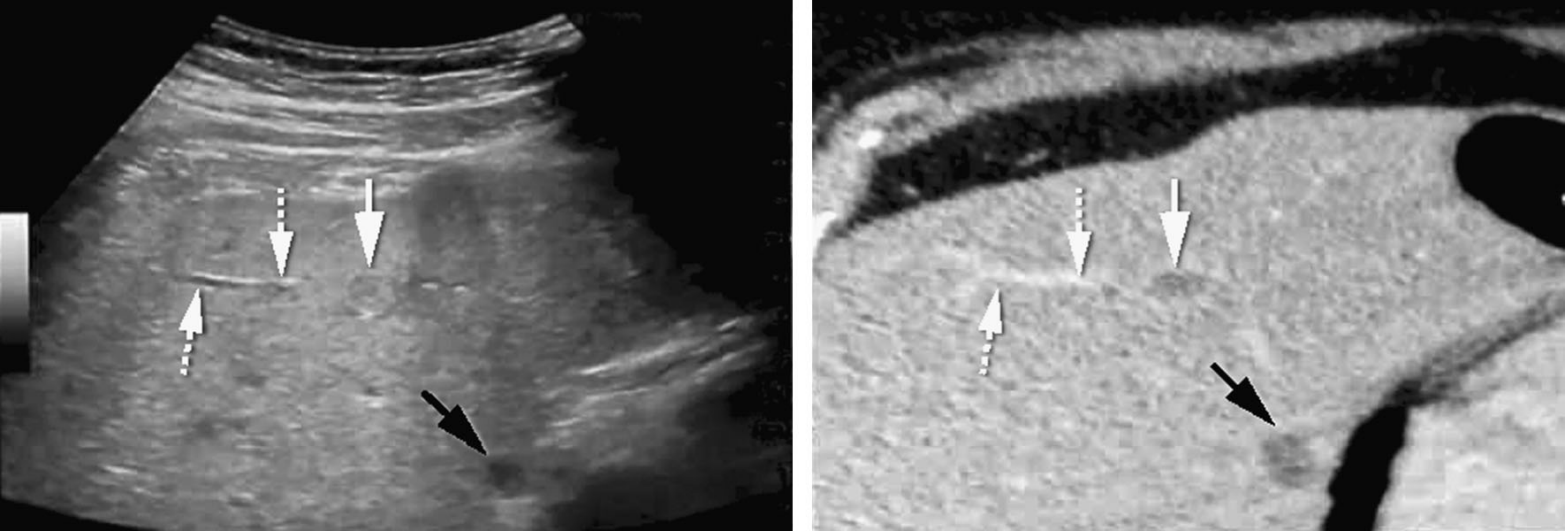
Example of automatic co-registration of US and CT images in a 65-year-old male with colorectal liver metastases. Two sub-centimeter lesions were characterized as metastases with the use of MRI (not shown), but were not found on pre-procedural ultrasonographic examination. The patient was scheduled to undergo ablation using the GE Logiq E9 navigation system. CT with intravenous contrast was obtained with the OmniTRAX™ Active Patient Tracker attached to the patient. The images show adequate co-registration of US (*left*) and CT (*right*) with matching position of a portal vein branch (*dotted arrows*) and liver cyst (*black arrows*). After image fusion, the liver metastasis were vaguely seen (*white arrow*; second lesion not shown) and could be targeted with a radiofrequency probe. Post-ablation CT showed a good location of the ablation zone, and no recurrence has occurred during follow-up

A General Electric Logiq E9 ultrasound system with XDclear platform (General Electric (GE) Healthcare, Wauwatosa, WI, USA) and multi-modality abdominal CIRS model 057 phantom (CIRS, Norfolk, VA, USA) were used to conduct the experiments. GE volume navigation software, a C1-6-D convex transducer and an electromagnetic signal transmitter (Ascension Technology, Shelburne, VT, USA) were used to allow fusion of US and CT images. An OmniTRAX™ Active Patient Tracker (CIVCO Medical Solutions, Kalona, IA, USA) was fixed on the anterolateral side of the phantom (Fig. 1). CT of the phantom was acquired using a Toshiba Aquilion 64 scanner (Toshiba Medical Systems, Otawara, Japan) with the following scanning parameters: tube voltage of 120 kVp, 1.0 mm slice thickness and in-plane resolution of 0.78 mm × 0.78 mm. The CT data were uploaded to a GE Logiq E9 ultrasound system (Fig. 1) prior to image fusion. Figure 2 illustrates the use of automatic co-registration in clinical practise.

### Measurement of Co-registration Accuracy

Several phantom experiments were conducted (see below). In each experiment, the accuracy of the co-registration method was determined. Accuracy was determined by measurement of the residual displacement by two independent observers (PM and CH). High accuracy corresponded to low residual displacement, i.e., low registration mismatch between the US and CT images. Inaccuracy referred to high residual displacement, i.e., large discrepancies between US and CT images. To measure the residual displacement, a marker was placed in the center of a lesion on the US images, i.e., center_US_. Then, the center of the lesion was identified on the CT images, i.e., center_CT_, and the distance between center_US_ and center_CT_ was measured in millimeters.

For manual co-registration methods, the root-mean-square deviation was recorded. The RMSD is an established method to quantify the reliability of image fusion, as it is the standard deviation of the mean distance between the corresponding registration points on CT and US. The RMSD for a set of *n* reference points is given by the formula:

$${\text{RMSD}} = \sqrt {\frac{{\sum\nolimits_{i = 1}^{n} {|\overrightarrow {x}_{{i,{\text{CT}}}} } - \overrightarrow {x}_{{i,{\text{US}}}} |^{2} }}{n}}$$

where $\overrightarrow {x}_{{i,{\text{CT}}}}$ and $\overrightarrow {x}_{{i,{\text{US}}}}$ are the position of the reference point *i* on CT and US, respectively.

### Experiments

#### Experiment A: Manual Versus Automatic Versus Semiautomatic Co-registration

**Fig. 3:**
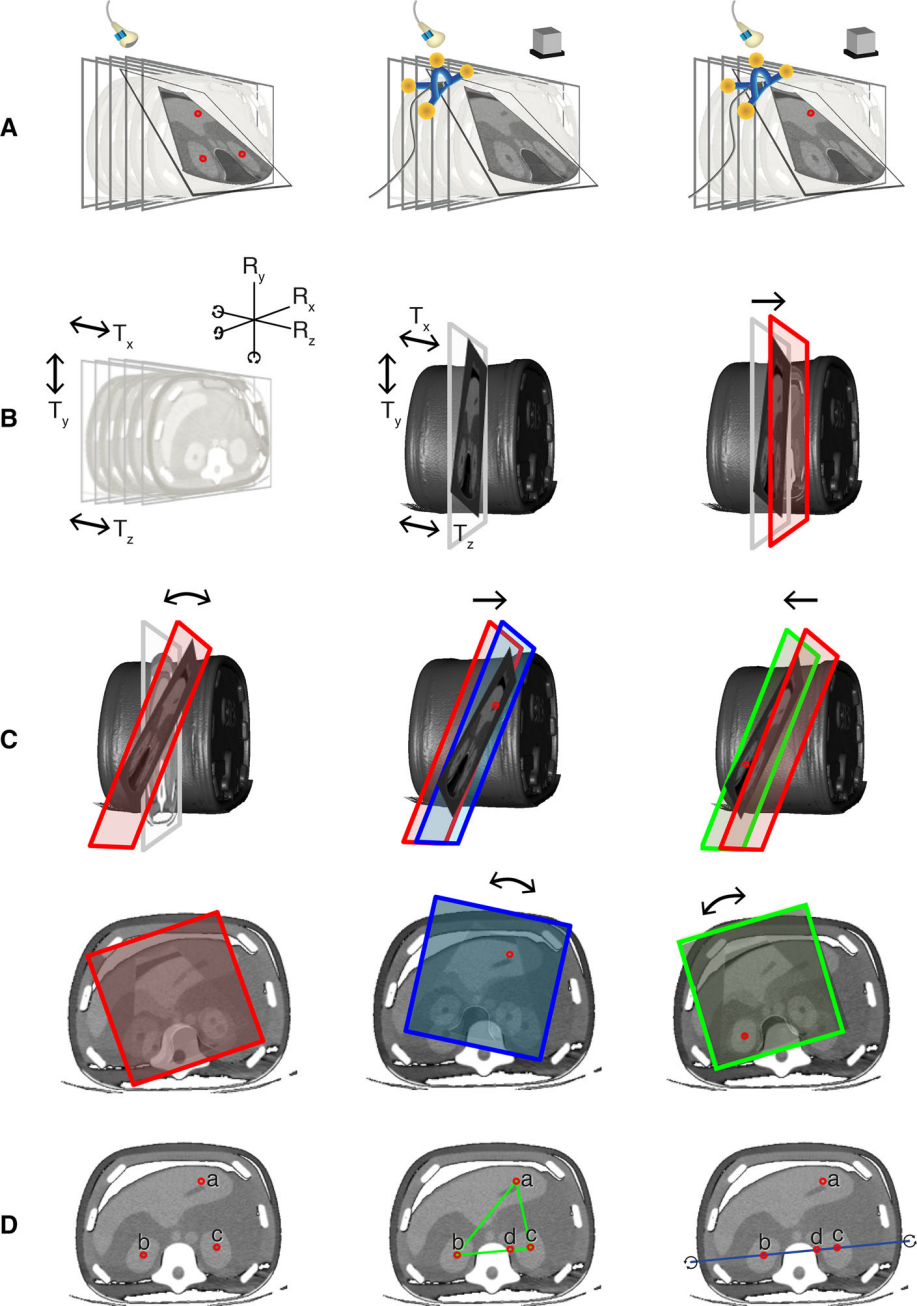
Graphical overview of the phantom experiments. **A** Comparison of manual point co-registration (*left*), automatic co-registration (*middle*) and semiautomatic co-registration (*right*). **B** Comparison of two manual co-registration methods: point co-registration (not shown; see A, *left*) and plane registration. Prior to plane registration, the orientation of the ultrasound plane could be changed by both rotation (Rx, Ry and/or Rz) and translation (Tx, Ty and/or Tz) (*left*). After fusion of the CT and US image by pressing the “lock plane” button on the US machine, correction of the image fusion was restricted to translational movements (*middle*). A single translation point was placed to optimize the co-registration (*right*). **C** Plane registration was conducted with deliberate mismatch between the CT and US planes. The US transducer was positioned at an angle of roughly 20° to the axial plane around the left–right axis (above) or at an in-plane rotation of roughly 20° around the feet-head axis (*below*). The angulated US plane was fused to an axial CT image. After this, correction of the co-registration was attempted by placing a well-identifiable point in the left hepatic vein (*middle*) and then in the center of the right kidney (*right*). **D** Comparison of manual selection of three reference points (*left*) or four reference points (*middle*) and automatic selection of three out of four reference points (*right*). As the left hepatic vein reference point (a) was deliberately placed 8 mm anteriorly, a system preference was enforced for the three reference points that were in line (as these resulted in the lowest RMSD). Rotation of the registration plane was restricted by the triangular orientation of the reference points (*middle*) in the experiments with operator-dependent point selection, whereas rotational errors around the blue line (*right*) occurred with automatic point selection

In the first experiment, the registration accuracy of manual point co-registration was compared with that of automatic co-registration and semiautomatic co-registration. Figure 3A provides a graphical overview of the different co-registration methods used in this experiment.

For manual point co-registration, three reference points were selected *manually* on both the US and CT images using the “point/all” registration option of the GE Logiq E9 system. The center of each kidney and a well-identifiable point of the left hepatic vein were chosen as reference points. Automatic co-registration was established using automatic detection of the active tracker within the electromagnetic field by the US system. Semiautomatic co-registration was realized by automatic co-registration and an additional translation correction by manual indication of a well-identifiable point in the left hepatic vein. Thus, automatic and semiautomatic co-registrations are similar except for the following: in semiautomatic co-registration, an additional reference point is placed manually after the automatic registration process to optimize the co-registration.

To compare the accuracy of the three different registration techniques, the residual displacement was measured for two different lesions in the phantom: a superficial target lesion at 50 mm from the surface and a target lesion at 80 mm from the surface. The co-registrations and measurements were repeated 20 times by each of the two observers.

#### Experiment B: Manual Point Registration Versus Plane Registration

In the second experiment, two methods of manual co-registration were compared (Fig. 3B). The first method was manual co-registration using three reference points as described above. In the second method, manual co-registration was established by so-called plane registration. After choosing an axial CT image, the phantom was scanned with the ultrasound probe in axial plane to find a matching US image. By pressing the “lock plane” button on the US machine, the US image was fused to the corresponding CT image. After this, correction of the image fusion was restricted to translational corrections. Then, a translation point was placed in order to optimize the co-registration.

The co-registrations and measurements were repeated five times by each of the two observers.

#### Experiment C: Pitfalls of Co-registration, Part I

The third experiment further examined co-registration using a plane (Fig. 3C). In this experiment, a deliberate mismatch was created between the CT and US plane. The transducer was positioned at an angle of roughly 20° to the axial plane around the left–right axis, while the CT images were maintained axial without angulation. Then, subsequent translation points were set to try to correct the registration mismatch: first at a well-identifiable point in the left hepatic vein and then in the center of the right kidney.

As the last part of this experiment, the transducer was carefully positioned axially on the phantom, but at an in-plane rotation of roughly 20° around the feet-head axis. The same two subsequent translation points were set as described above trying to correct the registration mismatch.

Each step of the experiment was repeated five times by each of the two observers with measurement of the registration accuracy for the superficial lesion and the center of the right kidney during each step.

#### Experiment D: Pitfalls of Co-registration, Part II

The last experiment examined manual co-registration using the “point/best3” option of the GE Logiq E9 system (Fig. 3D). This option allows automatic selection of reference points by the US system: When more than three reference points are manually selected by the operator, the US system automatically selects the three reference points that result in the lowest RMSD.

In the first step of this experiment, reference points were manually selected in the center of each kidney and at a well-identifiable point of the left hepatic vein. The left hepatic vein reference point was deliberately displaced 8 mm too far anteriorly in the sonogram to test a clinical scenario of operator-dependent misregistration. The second step was to evaluate whether the addition of a fourth reference would improve the co-registration accuracy. A fourth reference point was placed on the left edge of the spine, in line with the reference points in the kidneys. Finally, the “point/best3” option was selected on the US system to activate selection of the best three out of the four reference points by the US system based on RMSD calculations. As a result of the displacement of the middle hepatic vein reference point, a preference was enforced for automatic selection by the system of the three reference points that were in line.

After each step, the reported RMSD was recorded and the residual displacement was measured in the superficial lesion. All steps and measurements were repeated five times by each of the two observers.

### Statistical Analysis

Statistical analyses were performed using SPSS version 23.0 (IBM, Armonk, NY, USA). For all measurements, mean and standard deviation were derived as well as 95% confidence intervals (CI) of the mean. Using a two-way analysis of variance (ANOVA), the dependency of the accuracy on the position of the lesion and the co-registration method (manual using reference points, automatic and semiautomatic) was determined. Additionally, a one-way ANOVA was used to analyze the dependency of the accuracy on the position of the lesion for each of these co-registration methods separately. A one-way ANOVA was also used to determine the dependency of the registration accuracy on the number of reference points. A *p* value <0.05 was considered statistically significant.

## Results

For all co-registration experiments, measurements are listed in Table 1.

**Table 1:** Reported RMSD and residual displacement for different co-registration methods: mean, standard deviation and 95% CI of the mean

	Measure	Target	Mean ± SD (mm)	95% CI (mm)
*Experiment A*				
Manual point	RMSD		1.0 ± 0.4	0.8–1.1
	Residual displacement	Superficial lesion	2.4 ± 0.5	2.2–2.5
		Deep lesion	2.0 ± 0.6	1.9–2.2
Automatic	Residual displacement	Superficial lesion	5.9 ± 0.7	5.7–6.1
		Deep lesion	5.2 ± 0.6	5.0–5.4
Semiautomatic	Residual displacement	Superficial lesion	2.0 ± 0.7	1.8–2.3
		Deep lesion	2.5 ± 0.7	2.2–2.7
*Experiment B*				
Plane only	Residual displacement	Superficial lesion	13 ± 3	11–15
		Left kidney	13 ± 3	11–16
TP1: superficial	Residual displacement	Superficial lesion	1.9 ± 0.8	1.4–2.5
		Left kidney	4.5 ± 1.8	3.2–5.7
*Experiment C*				
20^0^ angulation around L-R axis				
Plane only	Residual displacement	Superficial lesion	17 ± 11	9–26
		Left kidney	33 ± 4	30–36
TP1: superficial	Residual displacement	Superficial lesion	4.7 ± 2.6	2.8–6.6
		Left kidney	34 ± 3	32–36
TP2: deep	Residual displacement	Superficial lesion	34 ± 3	32–36
		Left kidney	5.9 ± 1.8	4.6–7.2
20^0^ rotation around F–H axis				
Plane only	Residual displacement	Superficial lesion	35 ± 5	31–38
		Left kidney	54 ± 8	49–60
TP1: superficial	Residual displacement	Superficial lesion	18 ± 4	15–21
		Left kidney	26 ± 5	23–30
TP2: deep	Residual displacement	Superficial lesion	28 ± 6	24–32
		Left kidney	35 ± 7	30–40
*Experiment D*				
Three	RMSD		2.5 ± 0.5	2.1–2.8
	Residual displacement	Superficial lesion	6.2 ± 1.1	5.5–7.0
Four	RMSD		2.4 ± 0.5	2.0–2.7
	Residual displacement	Superficial lesion	5.5 ± 1.3	4.5–6.4
Best three of four	RMSD		0.9 ± 0.4	0.6–1.1
	Residual displacement	Superficial lesion	40 ± 26	21–58

*TP* translation point; *L–R* left–right; *F–H* feet–head; *RMSD* root-mean-square deviation

### Experiment A: Manual Versus Automatic Versus Semiautomatic Co-registration

**Fig. 4:**
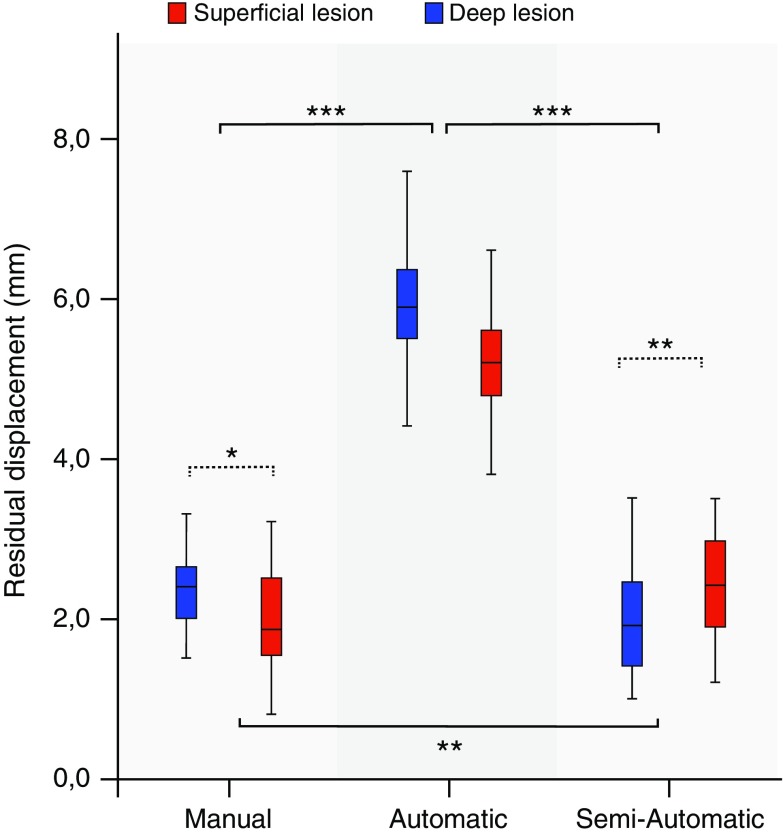
Comparison of manual, automatic and semiautomatic co-registrations. Accuracy is expressed as residual displacement between US and CT measured for a superficial lesion (*blue*) and a deep lesion (*red*). Centerlines in boxplots indicate the median; box edges indicate the 25th and 75th percentile. Uninterrupted brackets indicate comparisons between co-registration methods. Dotted brackets indicate comparison between lesions for a single co-registration method. *p < 0.05, **p < 0.01, ***p < 0.001

A significantly higher mean residual displacement was found with automatic co-registration compared to manual co-registration: 5.9 and 5.2 mm for the superficial and deep liver lesion, respectively, compared to 2.4 and 2.0 mm (Fig. 4). The accuracy of automatic co-registration improved significantly after applying a translation correction, i.e., semiautomatic co-registration (*p* < 0.0005). The residual displacement of semiautomatic co-registration was similar to the displacement found after manual co-registration: 2.0 and 2.5 mm for the superficial and deep lesion, respectively.

The accuracy depended on both the co-registration method and the position of the lesion. After manual co-registration, the mean displacement was significantly larger for the superficial lesion than for the deep lesion (*p* = 0.027). Conversely, the semiautomatic co-registration resulted in a larger displacement for the deep lesion than for the superficial lesion (*p* = 0.002).

### Experiment B: Manual Point Registration Versus Plane Registration

**Fig. 5:**
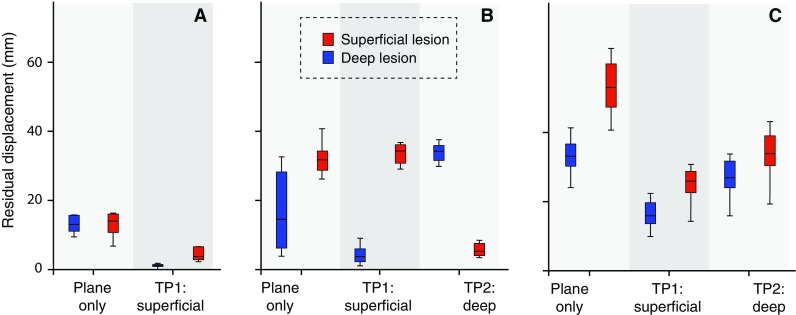
**A** Comparison of plane co-registration without (*right*) and with (*left*) additional translation point. Registration accuracy was measured for a superficial lesion (*blue*) and deep lesion (*red*). **B** Plane co-registration using a plane deliberately angulated around the left–right axis (*left*) resulted in poor registration accuracy with a wide range. Placement of a superficial translation point (TP1) resulted in improved registration accuracy for the superficial lesion, but not for the deep lesion (*middle*). Placement of a deep translation point (TP2) resulted in high registration accuracy for the deep lesion, but not for the superficial lesion (*right*). **C** Plane co-registration with a plane deliberately rotated around the feet-head axis resulted in poor registration accuracy with a wide range (*left*). Placement of a superficial translation point (TP1) or deep translation point (TP2) did not result in acceptable registration accuracies (*middle* and *right*)

After manual co-registration using a plane, a high residual displacement was found for both the superficial lesion and the left kidney (13.3 ± 3 mm for both). Upon placing a translation point, this accuracy improved to 1.9 ± 0.8 and 4.5 ± 1.8 mm, respectively (Fig. 5A).

### Experiment C: Pitfalls of Co-registration, Part I

Manual co-registration using a deliberately angulated plane resulted in poor registration accuracy with a wide range (Fig. 5).

Placement of a translation point did improve registration accuracy, but only for one of the two points of measurement (Fig. 5B). If the translation point was placed in the left hepatic vein, registration accuracy improved for the liver lesion but not for the center of the right kidney. If the translation point was placed in the right kidney, only the registration accuracy for center of the right kidney improved substantially.

After deliberate in-plane rotation of the US transducer around the feet-head axis, a substantial in-plane displacement was measured at both the superficial lesion and the right kidney (Fig. 5C). Again, assigning translation points led to an acceptable co-registration only near the most recently chosen translation point. Objects at other locations remained misaligned.

### Experiment D: Pitfalls of Co-registration, Part II

**Fig. 6:**
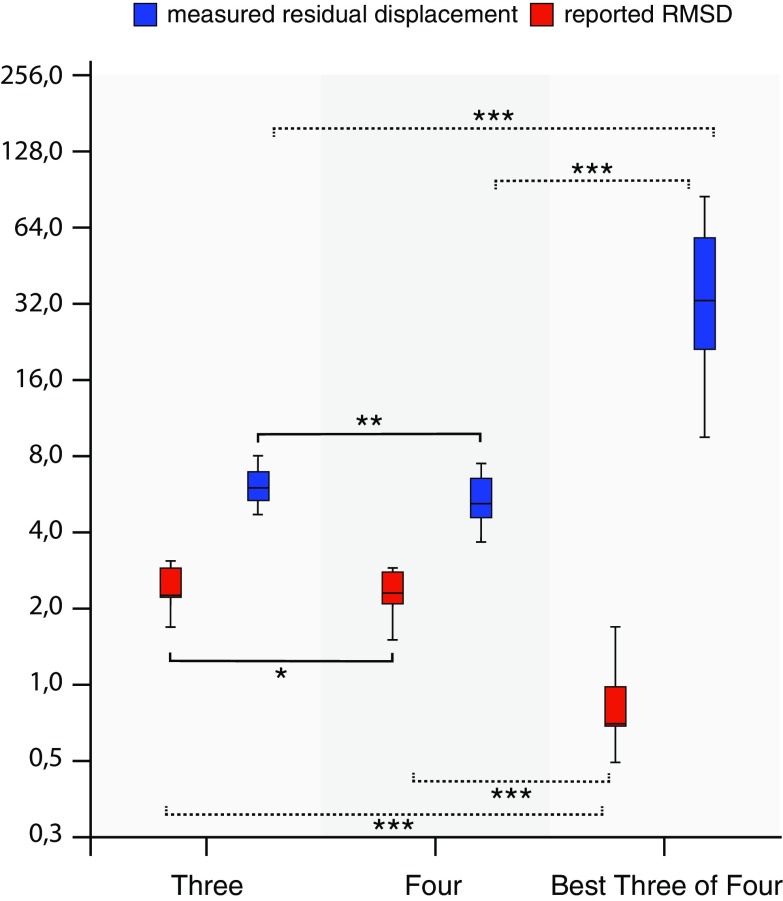
Comparison of manual point registration using three reference points (*left*), four reference points (*middle*) and software-based selection of three out of four reference points based on root-mean-square deviation (RMSD) (*right*). The registration error expressed as RMSD (*red*) does not correspond with the actual residual displacement (*blue*). Centerlines in boxplots indicate the median; box edges indicate the 25th and 75th percentile. Uninterrupted brackets indicate comparison of measured residual displacement for different co-registration methods. *Dotted brackets* indicate comparisons between reported RMSD. *p < 0.05, **p < 0.01, ***p < 0.001

Deliberate misplacement of one of the reference points during manual co-registration led to a high residual displacement in the superficial lesion (6.2 ± 1.1 mm) (Fig. 6). Adding a fourth reference point led to a nonsignificant (*p* = 0.91) improvement in registration displacement (5.5 ± 1.3 mm) (Fig. 6). Selection by the US system of the best three of four reference points resulted in a significantly worse residual displacement compared to using either three or four reference points (*p* < 0.0005, see Fig. 6). The mean reported RMSD, however, was significantly smaller in this case compared to the two co-registration methods with operator-dependent selection of reference points (*p* < 0.0005).

## Discussion

Basic knowledge of fusion technology and potential pitfalls is essential when using US systems with fusion imaging. We did not investigate the GE Logiq E9 navigation system in a clinical setting, so the implications of our phantom study for use of the system in patients are open to discussion. Nevertheless, it is likely that many of our study findings also apply in a clinical system. A co-registration method that is inaccurate in a phantom study is likely to have a higher co-registration mismatch in patients.

Our results demonstrate that automatic co-registration is significantly less accurate than manual co-registration when using the GE Logiq E9 navigation system (*p* < 0.0005). Based on our findings, we consider this registration method insufficient for routine use in clinical practice. The residual displacement of automatic co-registration was >5 mm. This increases the risk of technical failure (i.e., incomplete treatment or insufficient margins) in liver tumor ablation or of a sampling error in a percutaneous biopsy of a liver lesion. We therefore consider manual co-registration to be the preferred registration method. The accuracy of manual co-registration with the GE Logiq E9 has also been demonstrated in previous experiments, both in phantom studies as in healthy volunteers (2–10).

Semiautomatic co-registration is a valuable alternative in patients where manual registration is complicated by compromised ultrasonographic visibility and difficulties in identification of reference points. In our phantom study, the registration accuracy of semiautomatic co-registration was comparable to that of manual co-registration. Semiautomatic has an important disadvantage over manual co-registration. It requires acquisition of a contrast-enhanced CT or MRI just prior to the intervention with the tracker attached to the patient (same applies to automatic co-registration). This increases the procedure time as well as the radiation dose and contrast volume for the patient.

Based on our study findings, manual selection of reference points using the “point/all” mode offers the most accurate and reliable co-registration of the different manual co-registration methods of the GE Logiq E9 navigation system. Co-registration using a plane depends on the operator’s ability to identify an identical axial plane on the US images and the pre-intervention data. In clinical practice, matching the plane orientations in the first step of the registration process may be prone to errors as the positioning of the patient during the intervention may be different from that during the acquisition of the CT or MRI. As shown in our study, an initial mismatch between the CT and US plane cannot be sufficiently corrected by adding translation points. The addition of a translation point does shift the plane in the *X*-, *Y*- and/or *Z*-axis, but does not allow rotation of the plane. The registration accuracy may thus only be sufficient close to the intersection line between the US and CT planes. We therefore advise to use co-registration with plane registration with caution and only if placement of a translation point close to the target lesion is feasible.

From the current study, it was also found that assignment of reference points by the operator was more accurate than automatic selection of three reference points by the US system. The system’s selection algorithm is based on the lowest RMSD, which does not necessarily result in the best registration accuracy.

Similar to previous study findings, the current study shows that the accuracy is dependent on the position of the target lesion [9]. After manual co-registration, the residual displacement was slightly larger for the superficial target lesion than for the deep lesion. This is expected to be a direct consequence of the compression of the phantom by the transducer, which influences the position of a superficial lesion more than that of a deep lesion [13]. Conversely, semiautomatic co-registration was found to be less accurate for the deep target lesion than for the superficial lesion, which suggests that the accuracy decreases with increasing distance between the lesion and the active tracker. Preferably, both the active tracker and the translation point are placed close to the target lesion for improved accuracy.

Our study has several limitations. The performance of the US system in clinical practice may differ from the results obtained in our phantom study. Registration inaccuracies are expected to be greater in patients for all co-registration methods as motion, breathing and tissue compressibility may induce registration errors [14, 15]. Furthermore, patient positioning may have a negative impact on registration accuracy, as it may lead to increased mismatches due to deformation of tissue [13, 16, 17]. Another limitation of the study is that we only investigated the performance of the GE Logiq E9 and study findings may thus not be extrapolated to other systems and registration methods. Finally, reference points were chosen within the phantom kidneys for manual co-registration, because these could be identified more easily than other landmarks due to the limited anatomical detail in our phantom. In patients, reference points are preferentially placed within the liver when performing percutaneous liver interventions.

In conclusion, manual and semiautomatic co-registrations result in low registration inaccuracies in a phantom model and are preferred over fully automatic co-registration. Point registration is preferentially performed using all operator-assigned reference points rather than using automatic point selection by the US system. Plane registration is an alternative method, provided that the plane orientation is correctly chosen during the first step of the registration process.
